# An effective N6-methyladenosine-related long non-coding RNA prognostic signature for predicting the prognosis of patients with bladder cancer

**DOI:** 10.1186/s12885-021-08981-4

**Published:** 2021-11-21

**Authors:** Tianming Ma, Xiaonan Wang, Lingfeng Meng, Xiaodong Liu, Jiawen Wang, Wei Zhang, Zijian Tian, Yaoguang Zhang

**Affiliations:** 1 Department of Urology, Beijing Hospital, National Center of Gerontology, Institute of Geriatric Medicine, Chinese Academy of Medical Sciences, Beijing, 100730 P. R. China; 2 Graduate School of Peking Union Medical College, Chinese Academy of Medical Sciences, Beijing, 100730 P. R. China; 3 Department of Radiology, Beijing Hospital, National Center of Gerontology, Institute of Geriatric Medicine, Chinese Academy of Medical Sciences, Beijing, 100730 P. R. China

**Keywords:** Bladder cancer, N6-methyladenosine, Long non-coding RNA, Prognostic signature, Immune infiltration

## Abstract

**Background:**

Bladder cancer (BLCA) typically has a poor prognosis due to high relapse and metastasis rates. A growing body of evidence indicates that N6-methyladenosine (m6A) and long non-coding RNAs (lncRNAs) play crucial roles in the progression of BLCA and the treatment response of patients with BLCA. Therefore, we conducted a comprehensive RNA-seq analysis of BLCA using data from The Cancer Genome Atlas (TCGA) to establish an m6A-related lncRNA prognostic signature (m6A-RLPS) for BLCA.

**Methods:**

Consensus clustering analysis was used to investigate clusters of BLCA patients with varying prognoses. The least absolute shrinkage and selection operator Cox regression were used to develop the m6A-RLPS. The ESTIMATE and CIBERSORT algorithms were used to evaluate the immune composition.

**Results:**

A total of 745 m6A-related lncRNAs were identified using Pearson correlation analysis (|R| > 0.4, *p* < 0.001). Fifty-one prognostic m6A-related lncRNAs were screened using univariate Cox regression analysis. Through consensus clustering analysis, patients were divided into two clusters (clusters 1 and 2) with different overall survival rates and tumor stages based on the differential expression of the lncRNAs. Enrichment analysis demonstrated that terms related to tumor biological processes and immune-related activities were increased in patient cluster 2, which was more likely to exhibit low survival rates. Nine m6A-related prognostic lncRNAs were finally determined and subsequently used to construct the m6A-RLPS, which was verified to be an independent predictor of prognosis using univariate and multivariate Cox regression analyses. Further, a nomogram based on age, tumor stage, and the m6A-RLPS was generated and showed high accuracy and reliability with respect to predicting the survival outcomes of BLCA patients. The prognostic signature was found to be strongly correlated to tumor-infiltrating immune cells and immune checkpoint expression.

**Conclusions:**

We established a novel m6A-RLPS with a favorable prognostic value for patients with BLCA. We believe that this prognostic signature can provide new insights into the tumorigenesis of BLCA and predict the treatment response in patients with BLCA.

**Supplementary Information:**

The online version contains supplementary material available at 10.1186/s12885-021-08981-4.

## Background

Bladder cancer (BLCA), one of the most common types of cancer in the world, is associated with high mortality and a steadily rising morbidity worldwide [1]. Recent data have shown that among all cancers, BLCA had the fourth-highest incidence and eighth highest mortality in 2020, causing approximately 17,980 deaths in American men [2]. Although several therapeutic strategies, such as surgery and immune checkpoint inhibitors (ICIs), have been utilized to manage BLCA [3, 4], the prognosis for patients with BLCA remains poor due to the complex and heterogeneous properties of BLCA, resulting in a high frequency of post-treatment recurrence or metastasis [5, 6]. Accordingly, it is imperative to explore appropriate prognostic biomarkers and therapeutic targets of BLCA to improve clinical outcomes.

N6-methyladenosine (m6A), a reversible and abundant modification on messenger RNAs (mRNAs) and non-coding RNAs (ncRNAs), has been demonstrated to greatly affect various aspects of RNA metabolism, including splicing, stability, nuclear export, and translation [7]. Several studies have indicated that the aberrant expression of m6A regulators, which include the “writers” (methyltransferases), “readers” (binding proteins), and “erasers” (demethylases), can potentially induce m6A to actively participate in carcinogenesis, cancer development, and drug resistance in various types of cancer, including BLCA [7–9]. For instance, high expression of METTL3, an m6A methyltransferase, has been reported to be able to facilitate the proliferation and progression of hepatocellular carcinoma, colorectal cancer, and BLCA cells [10–12].

Previous studies have uncovered the influence of long non-coding RNAs (lncRNAs) on the regulation of various biological processes, including tumorigenesis and immunity [13, 14]. In a recent study, lncRNA SOX2OT overexpression was shown to contribute to the progression and poor prognosis of BLCA. SOX2OT knockdown resulted in the inhibition of BLCA cell growth, and SOX2 expression regulated by sponging miR-200c inhibited BLCA invasion [15]. In another study, lncRNA Gas6-AS2 was reported to act as an oncogenic lncRNA and a predictor of poor prognosis in patients with BLCA [16]. Mounting evidence supports the notion that the interaction between m6A and lncRNAs is involved in the growth and development of cancer [7, 17]. Thus, an m6A-related lncRNA-based prognostic model may be helpful in the understanding and management of BLCA. Here, we investigated the prognostic and immunologic significance of m6A-related lncRNAs and developed an m6A-related lncRNA prognostic signature (m6A-RLPS) to predict survival outcomes in patients with BLCA.

## Methods

### Data sources

Using The Cancer Genome Atlas (TCGA), we obtained transcriptome-sequencing (RNA-seq) information from 411 BLCA samples and 19 adjacent non-tumor samples with corresponding clinical data. Meanwhile, the expression matrices of 23 m6A-related genes (*METTL3*, *METTL14*, *METTL16*, *WTAPI*, *VIRMA*, *ZC3H13*, *RBM15*, *RBM15B*, *YTHDC1*, *YTHDC2*, *YTHDF1*, *YTHDF2*, *YTHDF3*, *HNRNPC*, *FMR1*, *LRPPRC*, *HNRNPA2B1*, *IGFBP1*, *IGFBP2*, *IGFBP3*, *RBMX*, *FTO*, and *ALKBH5*) were obtained based on the latest publications [8, 9]. In addition, we extracted gene expression profile and cinicopathological from GSE31189 dataset (tumor: *n* = 52, normal: *n* = 40), GSE31684 dataset (tumor: *n* = 93), and GSE51493 dataset (tumor = 12, normal = 3) in the Gene Expression Omnibus (GEO) database to validate the role of target lncRNAs,

### m6A-related lncRNAs identification

The lncRNA profile from TCGA was first screened based on the human reference genome (GRCh38.p12; https://www.ncbi.nlm.nih.gov/genome). Pearson correlation analysis was used to identify m6A-related lncRNAs (|Pearson R| > 0.4, *p* < 0.001). Subsequently, univariate Cox regression analysis was performed to determine prognostic m6A-related lncRNAs (*p* < 0.01). The Wilcoxon rank-sum test was used to compare the expression levels (visualized using heatmaps) of the prognostic m6A-related lncRNAs between tumor and normal tissues.

### Consensus clustering analysis

To further explore the expression characteristics of m6A-related lncRNAs in BLCA, we clustered the samples into different groups using the ConsensusClusterPlus R package. Survival analysis and the chi-square test or Fisher’s exact test were used to compare the survival rates between the clusters and to determine the relationships between the clinicopathological parameters and the clusters. Heatmaps were created using the pheatmap R package to visualize the differential expression of the m6A-related lncRNAs and clinicopathological parameters in the different groups.

### Gene set enrichment analysis (GSEA) and TIC profile

GSEA was conducted using the Hallmark, C2 KEGG v.7.1, and C7 v.6.2 gene sets, and GSEA v.4.0.3 (http://www.broadinstitute.org/gsea). The ESTIMATE algorithm was used to analyze the immune, stromal, and ESTIMATE scores for each sample. These scores represent the ratio of immune and stromal components and the total proportions of these components in the tumor microenvironment (TME) [18]. The CIBERSORT algorithm was used to estimate the abundance of tumor-infiltrating immune cells (TICs) [19]. Only tumor samples with *p* values < 0.05, in terms of quality filtering, were retained for subsequent analysis.

### Associations between TME scores and TICs

The Wilcoxon rank-sum test was used to determine the relationships between TME scores and TICs in the different clusters. Normal samples were removed, and some routine immune checkpoint molecules, including *CD274 (PD-L1)*, *CTLA4/CTLA- 4*, *LAG3/LAG-3*, *LGALS9 (GAL9)*, *HAVCR2 (TIM-3)*, *PDCD1 (PD-1)*, *PDCD1LG2 (PD-L2)*, and *TIGHT*, were selected to compare the expression differences in the clusters and investigate the correlation of these differences with the hub m6A-related lncRNAs.

### Establishment and validation of the prognostic signature

The entire cohort was randomly divided into training and validation groups at a cut-off of 1:1. The chi-square test or Fisher’s exact test was used to determine the differences between the clinical features of the training and validation groups. The m6A-related candidate lncRNAs strongly related to the overall survival (OS) were determined to establish an m6A-RLPS for BLCA using least absolute shrinkage and selection operator (LASSO) Cox regression analysis [20]. The formula risk core = $\sum \limits_{i=1}^n{Coef}_i\times {Expr}_i$ was used to calculate the risk score for each patient, where Coef_i_ is the coefficient and Expr_i_ is the expression value of the corresponding m6A-related lncRNAs.. Patients were classified into low- or high-risk groups according to the median risk score. Kaplan–Meier curves were then established to compare the survival between the groups. Time-dependent receiver operating characteristic (ROC) curves with values for the area under the curve (AUC) for the 1-, 3-, and 5-year OS rates were used to estimate the prognostic prediction efficiency of the m6A-RLPS. Heatmaps were generated to reveal the differential expression of the prognostic m6A-related lncRNAs in the low- and high-risk groups. Subsequently, univariate and multivariate Cox regression analyses were used to investigate the predictive value of the risk score and the clinicopathological parameters for the survival of patients with BLCA. Furthermore, survival analysis was performed to further elucidate the prognostic ability of the risk score in various subgroups stratified by age, sex, clinical stage, T stage, and N stage. Then, a nomogram was built based on significant information from the multivariate Cox regression analyses and further evaluated by the concordance index (C-index) and calibration curves.

Associations between the clinicopathological parameters, immune score, and m6A-RLPS risk level were evaluated using the chi-square test or Fisher’s exact test and are shown in heatmaps. The student’s *t*-test was used to determine the relationships between the risk scores and clinicopathological parameters, including age, sex, clinical stage, T stage, N stage, cluster, and immune score.

### Correlation between the m6A-RLPS and immune-related features

Correlations between the m6A-RLPS and immune cells were evaluated using the Wilcoxon rank-sum test and Spearman correlation analysis. The m6A-RLPS was also comprehensively analyzed to determine its relationship with some immune checkpoints in BLCA.

### Statistical analysis

All analyses were performed using R v.4.0.3 (http://www.R-project.org). Statistical significance was set at a two-tailed *p*-value < 0.05.

## Results

### Identification of prognostic and m6A-related lncRNAs in BLCA

**Fig. 1:**
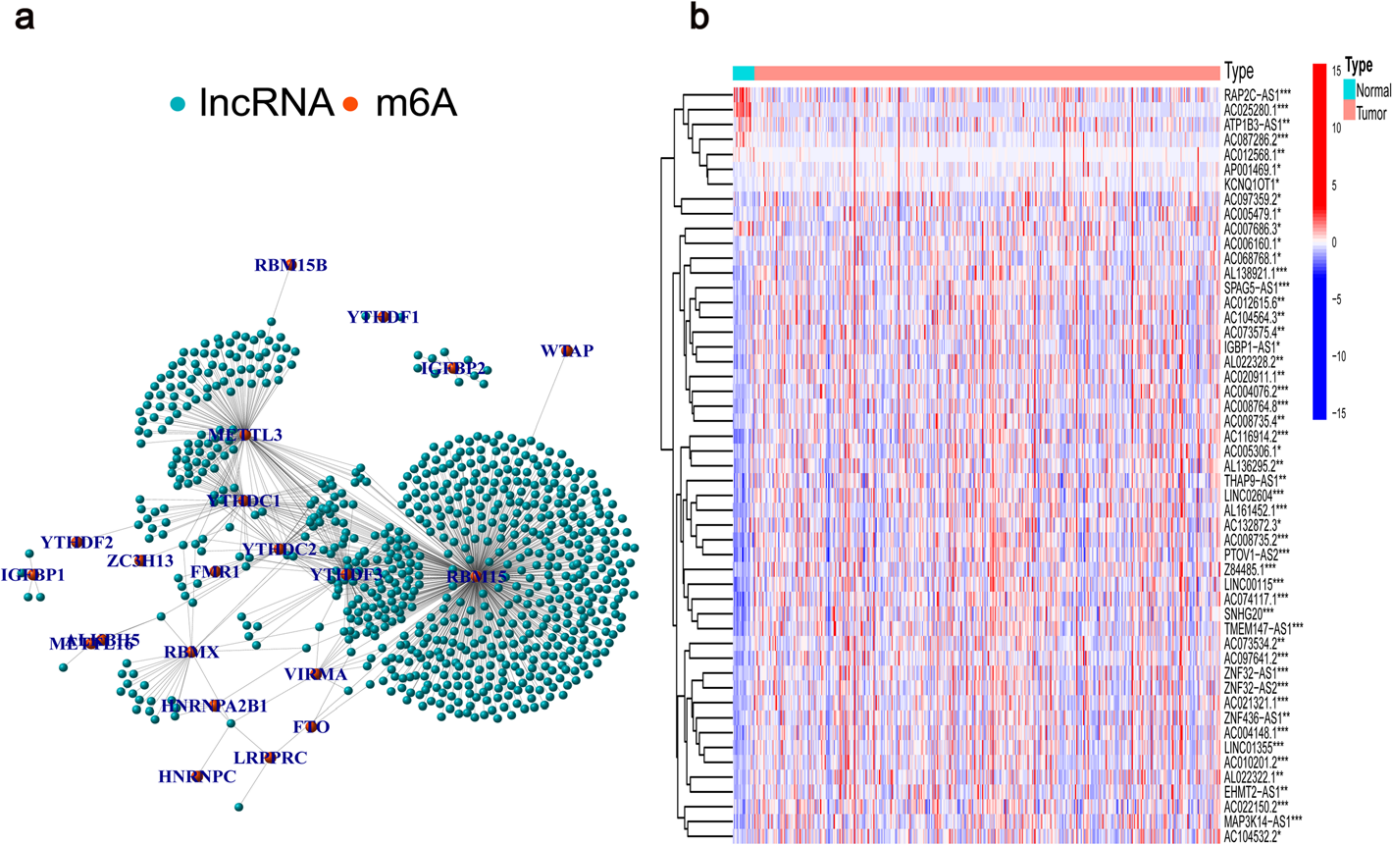
Screening of hub lncRNAs. (**a**) Network of the 23 selected m6A-related genes and their associated lncRNAs. (**b**) Differential expression of the 51 prognostic m6A-related lncRNAs in the normal and tumor samples. *p < 0.05, **p < 0.01, ***p < 0.001

A total of 14,086 lncRNAs were screened from TCGA data, and the detailed clinicopathological information of patients is shown in Additional file 1: Table S1. We determined that 745 m6A-related lncRNAs were significantly correlated with 23 m6A-related genes using Pearson correlation analysis (Fig. 1a). After excluding patients without cancer or survival data, we merged the survival data with the lncRNA expression data of individual patients (final patient number = 403). Subsequently, we identified 51 lncRNAs related to OS (Additional file 2: Table S2). Figure 1b shows that the expression of these prognostic m6A-related lncRNAs differed significantly between normal and BLCA tissues.

### Consensus clustering of m6A-related lncRNAs identified in molecular subtypes of BLCA

**Fig. 2:**
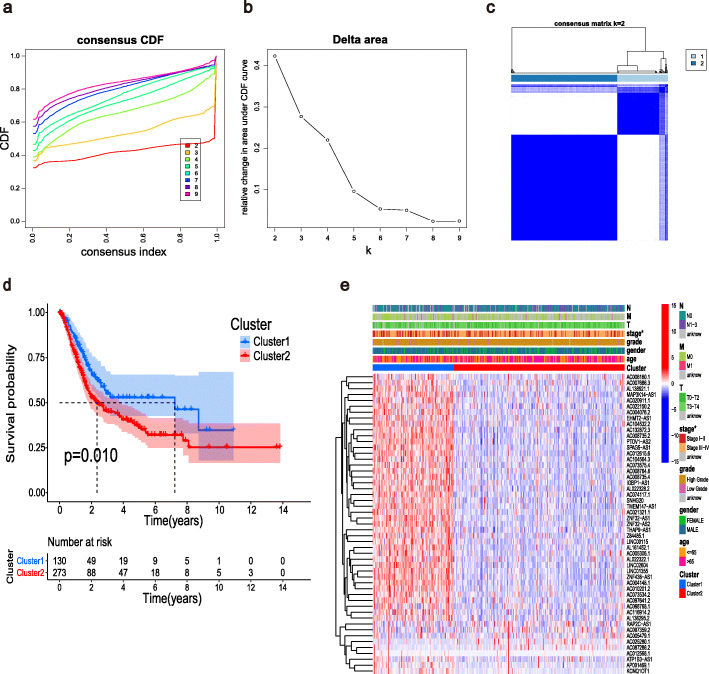
Consensus clustering analysis for the m6A-related lncRNA expression. (**a**) Consensus clustering cumulative distribution function (CDF) for k = 2 to 9. (**b**) Relative change in area under the CDF curve for k = 2 to 9. (**c**) Consensus matrix for k = 2. (**d**) Kaplan–Meier survival analysis for the two clusters. (**e**) Relationships between the m6A-related lncRNA expression and the clinicopathological parameters. *p < 0.05

Based on the expression profile of the prognostic m6A-related lncRNAs, we categorized the patients with BLCA into two groups: cluster 1 (*n* = 130) and cluster 2 (*n* = 273) (Fig. 2a–c). Survival analysis demonstrated that patients in cluster 2 had a worse OS than those in cluster 1 (*p* = 0.010, Fig. 2d). Figure 2e shows the relationships between the two clusters, revealing a distinct difference between the clusters in terms of the tumor stage (*p* < 0.05). Thus, the results of the consensus clustering analysis are associated with the progression of BLCA and survival of patients with BLCA.

### GSEA and immune-related analysis of the two clusters

**Fig. 3:**
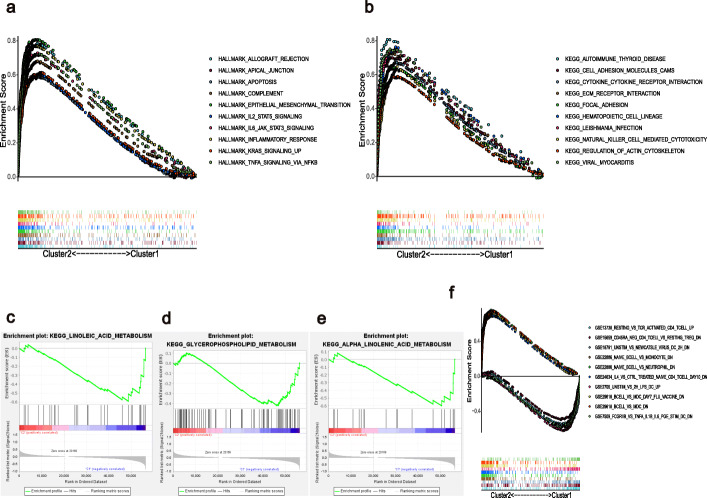
Gene set enrichment analysis for clusters 1 and 2. (**a**) Tumor hallmarks were enriched in cluster 2. C2 KEGG collection for (**b**) cluster 2 and (**c**–**e**) cluster 1. (f) Multi-GSEA enrichment curves for the C7 collection for clusters 1 and 2

The results of GSEA show that terms associated with multiple tumor hallmarks, such as apoptosis, epithelial-mesenchymal transition, inflammatory response, TNFA signaling, and IL6-JAK-STAT3 signaling, were predominantly enriched in cluster 2 (NOM p and FDR q-value < 0.001, Fig. 3a). C2 KEGG analysis similarly revealed several tumor-related signaling pathways and cell adhesion activities in cluster 2, whereas only three gene sets were significantly enriched in cluster 1, with metabolic pathways at a NOM *p*-value < 0.05 (Fig. 3b–e). Surprisingly, several enriched immune-related signaling pathways and genes were identified through both Hallmark and C2 KEGG analyses. C7 collection analysis indicated that multiple immune functional gene sets were enriched in clusters 1 and 2 (Fig. 3f).

**Fig. 4:**
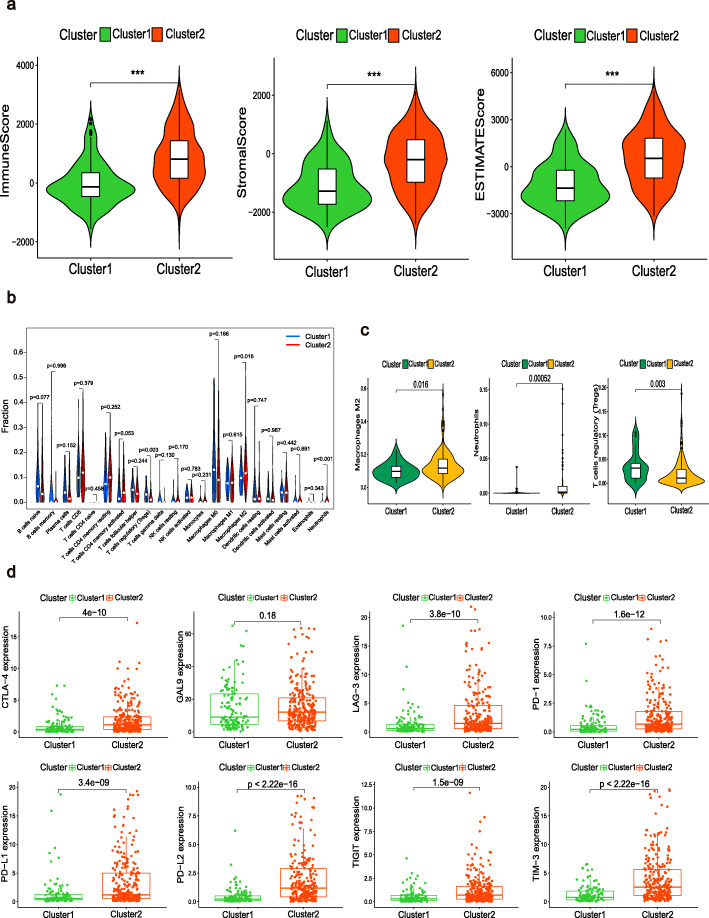
Immune status of the two clusters. (**a**) Violin plots depicting the differences in the immune score, stromal score, and ESTIMATE score of the two clusters. ***p < 0.001. (**b**) Proportions of the 22 types of immune cells in BLCA samples with different clusters. (**c**) Identification of three types of TICs with *p* < 0.05. (d) Distribution of eight immune checkpoints

Interestingly, we found a significant difference between the scores in clusters 1 and 2 (Fig. 4a). In addition, among the TICs in the BLCA, regulatory T cells (Tregs), neutrophils, and M2 macrophages were significantly associated with the clusters (Fig. 4b–c). These findings suggest that the expression of m6A-related lncRNAs in the clusters might have immunomodulatory effects on the TME. Given this, we attempted to determine the correlation between the clusters and some immune checkpoints. It is worth noting that seven types of immune checkpoint molecules, except for *GAL9*, were highly expressed in cluster 2 (Fig. 4d). The correlations between the 51 lncRNAs and the eight immune checkpoint molecules are shown in Additional file 3: Fig. S1 and most of these correlations were significant. Based on these results, we speculate that the poor prognosis in cluster 2 was probably due to the upregulation of the immune checkpoint molecules.

### Construction and validation of the m6A-RLPS

**Fig. 5:**
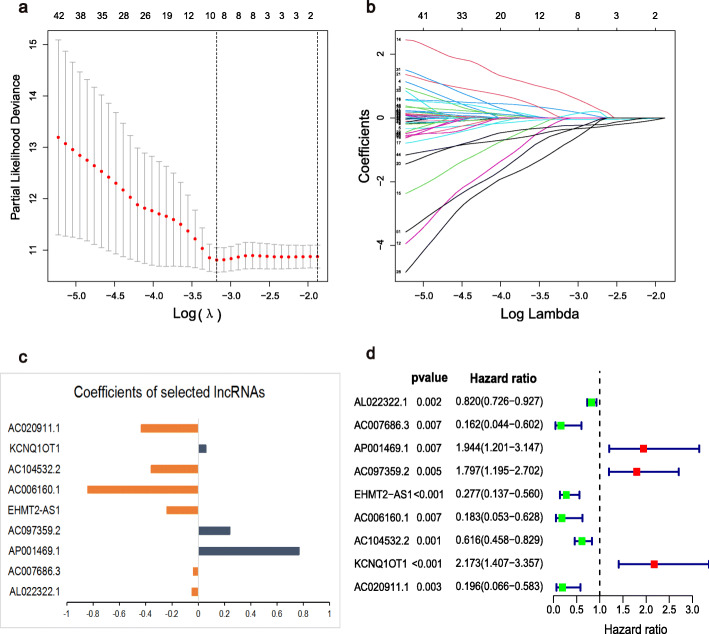
Establishment of the m6A-RLPS. (**a**–**c**) LASSO Cox regression analysis determined nine m6A-related lncRNAs and their corresponding coefficients; (d) Forest plot of the univariate Cox regression analysis demonstrating nine m6A-related lncRNAs

There were no significant differences between the training cohort(*n* = 203) and validation cohort (*n* = 200) in terms of any of the clinicopathological parameters (Additional file 4: Table S3). Next, LASSO Cox regression analysis incorporated nine prognostic m6A-related lncRNAs to the m6A-RLPS for predicting the OS of patients with BLCA (Fig. 5a–c). The risk score for each patient was calculated as follows: risk score = (**−** 0.433 × AC020911.1 expression) + (0.059 × KCNQ1OT1 expression) + (**−** 0.359 × AC104532.2) + (**−** 0.841 × AC006160.1) + (**−** 0.239 × EHMT2-AS1 expression) + (0.242 × AC097359.2 expression) + (0.768 × AP001469.1 expression) + (− 0.039 × AC007686.3 expression) + (− 0.048 × AL022322.1). Univariate Cox regression analysis supported the remarkable prognostic significance of all nine lncRNAs, among which AC097359.2, AP001469.1, and KCNQ1OT1 were identified as risk factors with a hazard ratio (HR) > 1, whereas the others were found to be protective factors (all *p* values < 0.01, Fig. 5d).

Next, we collected the expression data from the GEO DataSets platform for validation analysis of the 9 lncRNAs. Ultimately, only KCNQ1OT1 among the m6A-RLPS was detected in the GSE31189, GSE31684, and GSE51493 cohorts for subsequent analyses. The results showed that KCNQ1OT1 was markedly overexpressed in BLCA samples compared with normal tissues or in the high pathological T stage. Furthermore, Kaplan-Meier log-rank test supported the KCNQ1OT1 was a remarkable prognostic risk factor for BLCA in GSE31684 (Additional file 5: Fig. S2). These findings were in accordance with the results of TCGA analysis.

**Fig. 6:**
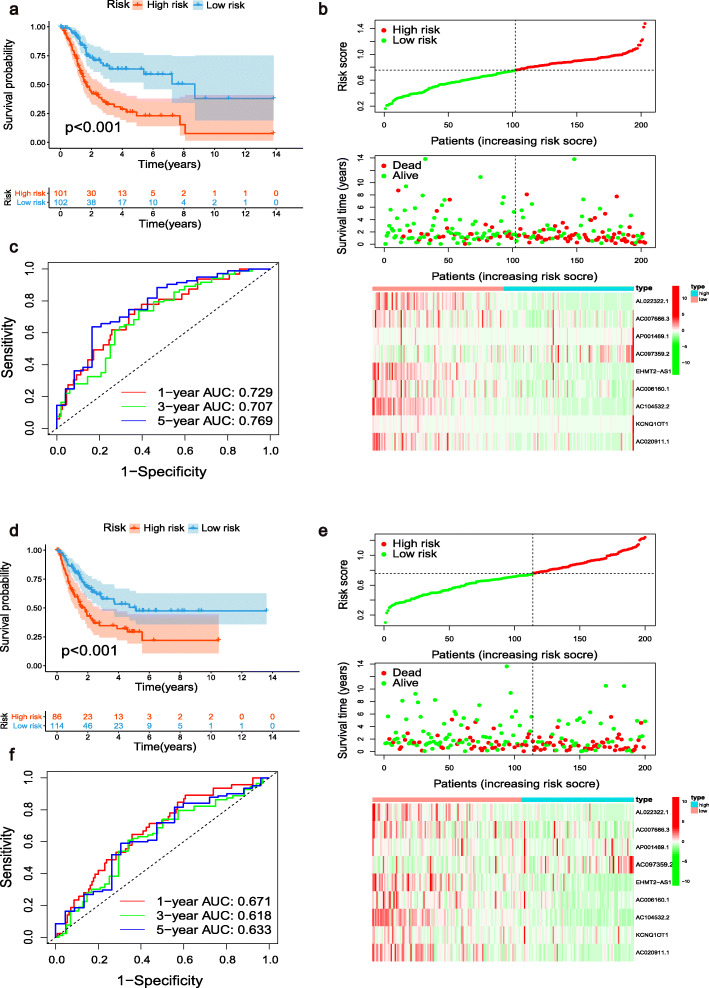
Analysis of the m6A-RLPS stratified by risk level. Kaplan–Meier curves for the m6A-RLPS in the (**a**) training and (**d**) validation cohorts. Distributions of risk scores, survival status, and relative lncRNA expressions in the (b) training and (**e**) validation cohorts. ROC curves for predicting 1-, 3-, and 5-year OS rates in the (**c**) training and (**f**) validation datasets

Subsequently, Kaplan–Meier curves showed that in the training cohort, patients in the low-risk group had an improved OS compared with those in the high-risk group (*p* < 0.001, Fig. 6a). Similar results were obtained in the validation cohort and the entire cohort (both *p* < 0.001, Fig. 6d, Additional file 6: Fig. S3a). The distributions of risk score, survival status, and corresponding lncRNA expression in each cohort are shown in Fig. 6b, Fig. 6e, and Additional file 6: Fig. S3b, respectively. The ROC curves showed that the m6A-RLPS could accurately forecast the OS in the training cohort, and the AUCs were 0.729, 0.707, and 0.769 for the 1-, 3-, and 5-year OS rates, respectively (Fig. 6c). Similar results were subsequently verified in the validation cohort and the entire cohort (Fig. 6f, Additional file 6: Fig. S3c). These findings indicate that the prognostic signature has a robust and stable predictive efficiency.

### The m6A-RLPS was an independent prognostic indicator in BLCA

**Fig. 7:**
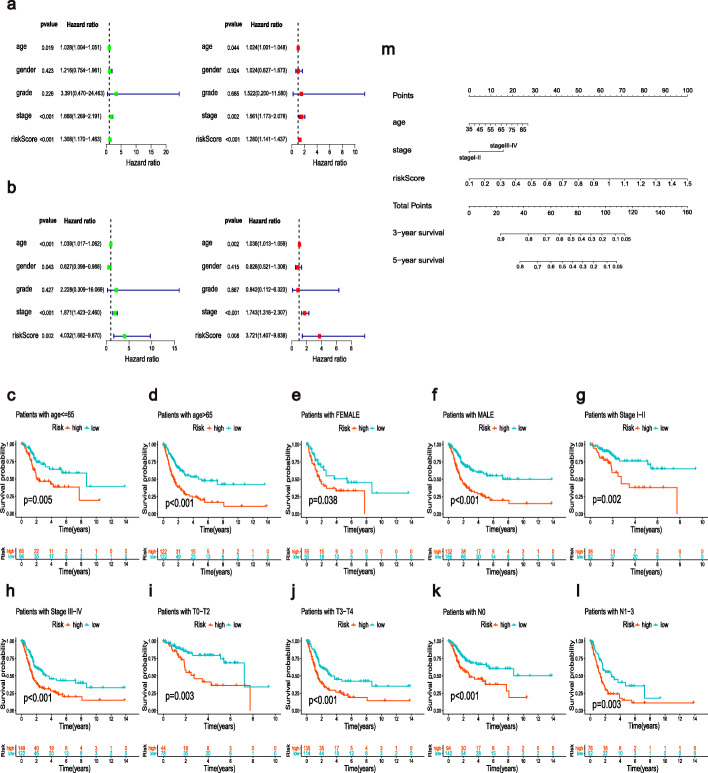
The m6A-RLPS is an independent prognostic indicator. Univariate and multivariate Cox regression analysis in the (**a**) training and (**b**) validation cohorts simultaneously demonstrated the independent prognostic value of the risk score. Survival analysis stratified by (**c**, **d**) age, (**e**, **f**) gender, (**g**, **h**) clinical-stage, (**i**, **j**) T stage, and (k,l) N stage. (m) Nomogram based on age, tumor stage, and risk score in the training cohort

The result of the univariate analysis indicated that age (*p* < 0.01), tumor stage, and risk score (both p < 0.001) were closely associated with prognosis (Fig. 7a). Multivariate analysis validated that age, tumor stage, and risk score were independent prognostic factors in patients with BLCA (Fig. 7b). Stratification survival analysis showed that high-risk patients had an observably worse OS than low-risk patients in every subgroup (Fig. 7c–l), indicating a notable predictive performance of the m6A-RLPS. Nomograms for the 3- and 5- year OS rates based on the independent predictors determined from the multivariate analysis are shown in Fig. 7m. A certain point was generated for each covariate, and a total nomogram score, which was correlated with the 3- and 5-year OS rates, was calculated for every patient. The nomogram showed favorable accuracy in predicting the OS, with a C-index of 0.71 (95% CI: 0.61–0.77) and 0.68 (95% CI: 0.62–0.74) for the training and validation cohorts, respectively. Moreover, the calibration curves revealed that there was an appreciable agreement between the predictive outcome and actual survival, and similar conclusions were obtained in the validation cohort (Additional file 7**:** Fig. S4). In addition, we further determined the utility of the m6A-RLPS risk score across 33 kinds of TCGA cancer using the transcriptome and clinicopathological data acquired from the UCSC Xena project (http://xena.ucsc.edu). The univariate Cox regression results showed that the m6A-RLPS risk score was significantly related to OS in 9 types of cancer (BLCA, COAD, KICH, KIRC, LGG, PAAD, SKCM, STAD, and UVM). Among them, the m6A-RLPS risk score was a risk factor in BLCA, PAAD, SKCM, and STAD (HR > 1, *p* < 0.05), whereas all others were protective factors. Considering the possibility of death from non-tumor causes during follow-up, we also analyzed the relationship between the m6A-RLPS risk score and Disease-specific survival (DSS) in 33 TCGA tumors. The univariate Cox regression results also showed that the m6A-RLPS risk score was a risk factor in BLCA, PAAD, OV, SKCM, and UCSE, but was a protective factor in KIRC, LGG, and UVM (Additional file 8**:** Table S4). These results demonstrated the predictive value of the m6A-RLPS for some other cancers.

### The m6A-RLPS was correlated with clinicopathology and TME immune activity

**Fig. 8:**
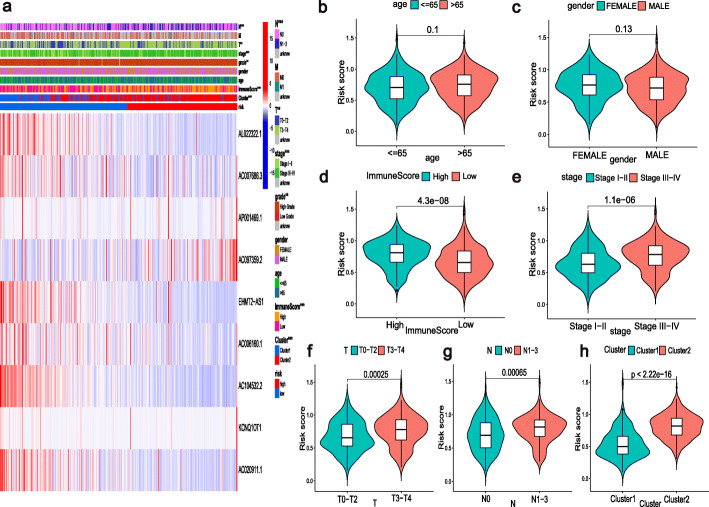
Relationships between the risk score and the clinicopathological parameters. (**a**) Heatmap of the chi-square test or Fisher’s exact test illustrating the associations between the m6A-RLPS risk level and the clinicopathological parameters. **p < 0.01, ***p < 0.001. Distribution of risk scores according to (**b**) age, (**c**) gender, (**d**) immune score, (**e**) clinical-stage, (**f**) T stage, (**g**) N stage, and (**h**) cluster

Figure 8a shows that the expression of lncRNAs included in the m6A-RLPS was significantly correlated with cluster type, immune score, tumor grade, tumor stage, T stage, and N stage (all *p* values < 0.01). In addition, the Student’s *t*-test demonstrated that the risk score increased with increasing immune score, clinical stage, T stage, and N stage (Fig. 8d–g) and that high-risk patient tended to be gathered in cluster 2 (Fig. 8h), but the risk score was not significantly correlated with age and sex (Fig. 8b-c). These findings suggest that the m6A-RLPS can influence the progression of BLCA.

**Fig. 9:**
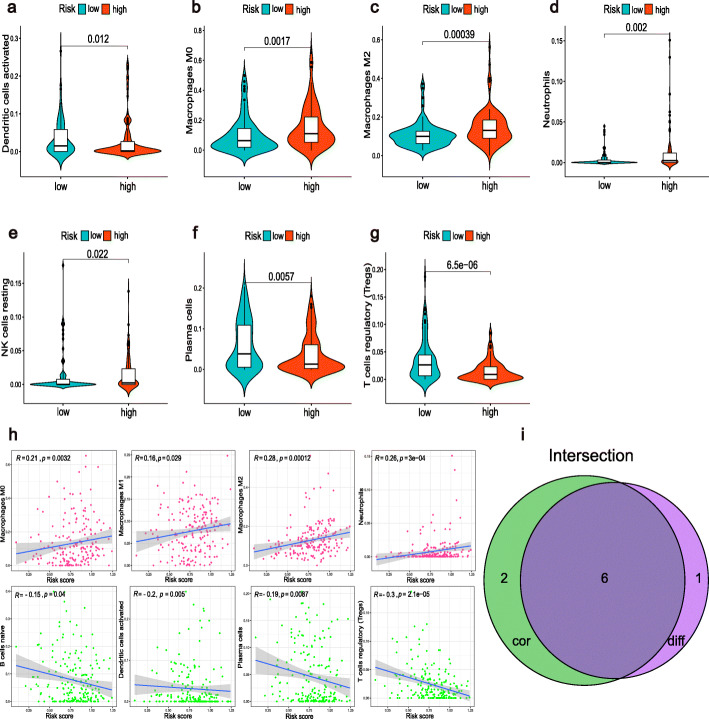
The m6A-RLPS is associated with TICs. (**a**–**g**) Violin plots of the difference analysis confirmed seven types of TICs. All *p* values < 0.05. (**h**) Correlation analysis determined eight types of TICs. (**i**) Venn diagram of common TICs

**Fig. 10:**
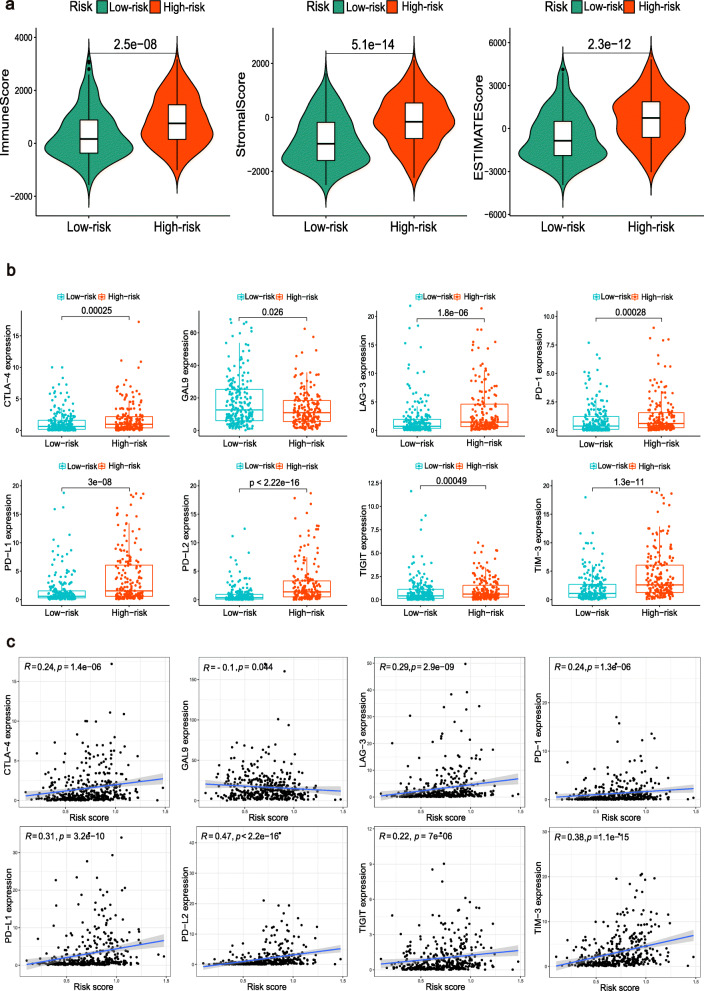
The m6A-RLPS is related to TME immune reaction. (**a**) Distribution of TME scores in the risk groups. (**b**) Differential expression of eight immune checkpoints in the risk groups. (**c**) Nearly all the selected immune checkpoints positively correlated with the risk score

By combining the difference and correlation analyses, we found that six types of TICs, including plasma cells, Tregs, M0 macrophages, M2 macrophages, activated dendritic cells, and neutrophils were strikingly associated with the m6A-RLPS (Fig. 9). Of these associations, three showed positive correlations (M0 macrophages, M2 macrophages, and neutrophils), whereas the others showed negative correlations. In addition, we discovered that the m6A-RLPS also had a strong positive correlation with the TME scores obtained using the ESTIMATE algorithm (Fig. 10a). We also noticed that the expressions of immune checkpoints, except for *GAL9*, were increased in high-risk patients and positively correlated with the risk score, reflecting the effect of the immune checkpoints on TME and poor oncological outcomes (Fig. 10b–c).

## Discussion

BLCA is a complex and heterogeneous tumor that is associated with high morbidity and poor prognosis [1, 5]; thus, the development of a reliable prognostic model for BLCA with a satisfactory predictive capability is important. With the development of sequencing and other technologies, genetics-based molecular subtyping of BLCA has been increasingly investigated, providing more tumor biological information than the traditional classification system [21]. Moreover, recent studies have addressed the significance of the m6A modification and lncRNAs in the development and progression of urological cancer [22, 23], and several prognostic signatures based on m6A regulators or lncRNAs alone have been identified [24, 25]. However, to the best of our knowledge, an accurate and applicable prognostic signature based on m6A-related lncRNAs for patients with BLCA has not been identified.

Accordingly, we comprehensively analyzed the RNA-seq data of BLCA hosted on TCGA. A total of 745 m6A-related lncRNAs were identified, and 51 of them were determined to be of prognostic value. Additionally, we defined two clusters by consensus clustering analysis to investigate the potential molecular subtypes of BLCA. The results indicate that the cluster subtypes were strongly linked to the tumor stage and OS, and cluster 2 had a worse OS and higher clinical stage than those in cluster 1, reflecting the associations between m6A-related lncRNAs and the progression and prognosis of BLCA. Numerous studies have demonstrated that m6A can interact with lncRNAs to affect tumorigenesis and metastasis through a variety of mechanisms; however, these mechanisms remain unclear in the context of BLCA progression. In particular, METTL3-mediated m6A modification was found to stabilize the lncRNA LINC00958 transcript to increase the expression of the hepatoma-derived growth factor, ultimately facilitating the growth of hepatocellular carcinoma [26]. He et al. reported that ALKBH5 inhibited the progression of pancreatic cancer by stabilizing lncRNA KCNK15-AS1 [27]. Furthermore, lncRNA GAS5-AS1 has been shown to suppress the proliferation of cervical cancer cells by interacting with ALKBH5 [28]. These findings validate the functions and roles of lncRNAs and m6A in tumors, providing insights for understanding the mechanisms underlying the development and progression of BLCA. As such, we attempted to further elucidate the functions of the differentially regulated m6A-related lncRNAs in the two clusters via GSEA, and we observed that cluster 2 was associated with malignancy-related signaling pathways. Moreover, terms related to the numerous immune-related activities enriched in the two clusters revealed that the m6A-related lncRNAs were related to immune function. The results of GSVA revealed that the angiogenesis-related pathway, immune response-related pathway, etc. which are considered immunosuppressive and play a vital role in tumorigenesis were enriched in cluster 2.

The functional annotation was entirely consistent with the survival analyses that cluster 2 had a worse OS than those in cluster 1. Consistent with our findings, recent studies have shown that both m6A and lncRNAs play pivotal regulatory roles in the immune system, especially in immune activation and immune cell infiltration [7, 14, 29]. Based on these findings, we obtained the TME score and immune landscape of each BLCA sample to investigate the relationships among the clusters, TME, and immune checkpoints. We found that the TME scores, three types of TICs (Tregs, neutrophils, and M2 macrophages), and seven immune checkpoints were significantly different between the two clusters. Among them, cluster 2 had a higher immune score than cluster 1, meanwhile, neutrophils, M2 macrophages, and immune checkpoint molecules (*PD-L1, CTLA- 4*, *LAG-3*, *TIM-3, PD-1, PD-L2,* and *TIGHT*) were highly expressed in cluster 2, emphasizing that the molecular subtypes in the study based on the expression pattern of m6A-related lncRNAs is closely associated with immunity and oncogenesis [30–32]. These results also demonstrated that the above molecular subtypes exist independent of pathological stage stratification, conferring the molecular subtypes as intrinsic tumor features, and the molecular phenotyping might have a significant influence on behavior and treatment response of the tumor compared to pathological stratification.

Among the 51 m6A-related lncRNAs, 9 lncRNAs were used to generate the m6A-RLPS, which stratified BLCA patients into low- and high-risk groups with distinct OS and exhibited considerably good performance. Univariate and multivariate Cox regression analyses showed that the m6A-RLPS was an independent prognostic factor for OS. We validated the predictive capacity of our prognostic signature in patients stratified based on the clinicopathological parameters. We noticed that the m6A-RLPS exhibited a strong positive correlation with the clinicopathological parameters, including the T and N stages in BLCA. Moreover, by integrating the m6A-RLPS, age, and tumor stage, we were able to construct a quantitative nomogram, which was highly accurate and reliable with respect to estimating the survival of individuals.

It is worth noting that lncRNA KCNQ1OT1 had the strongest association with poor survival based on our prognostic signature (HR = 2.173, 95% CI: 1.407–3.357, *p* < 0.001). Some investigators have suggested that KCNQ1OT1 can facilitate cell proliferation, invasion, and metastasis in multiple types of cancers, such as prostate cancer, hepatocellular carcinoma, and osteosarcoma [33–35]. In particular, Li et al. reported that KCNQ1OT1 promoted BLCA progression by targeting miR-218-5p/HS3ST3B1 [36]. Similarly, Wang et al. found that KCNQ1OT1 might accelerate cell proliferation and migration in BLCA by regulating the miR-145-5p/PCBP2 axis [37]. These findings, which validate the oncogenic property of KCNQ1OT1, are in line with our results. Unfortunately, there are currently very few studies on the remaining eight lncRNAs. Therefore, we expect that our results will assist in demonstrating the prognostic value of these m6A-targeted lncRNAs, thereby offering insights into their potential roles in the oncogenesis and progression of BLCA.

There is increasing evidence regarding the clinical significance of the TME in the context of predicting tumorigenesis, progression, prognosis, and therapeutic efficacy in various cancers; TICs in the TME play an important role in these processes [38, 39]. As mentioned, we confirmed that the m6A-related lncRNAs in the clusters play vital roles in determining the immune status in BLCA. Thus, we conducted a thorough analysis of the association between the m6A-RLPS and TME immune activity. The results showed that the risk scores were closely related to the TME scores. Additionally, we compared the TICs between the high- and low-risk groups and found that seven types of immune cells were differentially present in BLCA. Correlation analysis showed that a high-risk score correlated with a high M2 macrophage level and low Treg level. M2 macrophages and Tregs are reported to be associated with tumorigenesis, progression, and immunotherapy [40, 41]. Recently, many studies have focused on immune checkpoint molecules, such as CTLA-4 and PD-1/PD-L1, as components of new strategies for cancer therapy, and found that these molecules can significantly regulate the immune function of TICs [41, 42]. BLCA is immune-responsive, and the efficacy and safety of immunotherapy in peri-operative settings in non-metastatic BLCA are being assessed in several trials [43]. Furthermore, m6A RNA modification is gaining increasing attention as a potential determinant of therapeutic resistance, including immunotherapy resistance within various cancers, and several lncRNAs have also been shown to affect the outcomes of immunotherapy [32, 44, 45]. In our study, the expression of almost all the immune checkpoints was positively correlated with the risk score predicted by our prognostic signature, suggesting the potential role of our m6A-RLPS in estimating the response to ICIs.

In this study, we employed and analyzed sufficient clinical and survival data from patients with BLCA. However, this study has several limitations. The retrospective design of our study allowed for the existence of confounding factors. Furthermore, because of the lack of BLCA samples and large independent clinical data, we were not able to validate the findings clinically. In addition, we did not examine the detailed roles of the other eight m6A-related lncRNAs in BLCA or determine how these m6A-related lncRNAs participate in BLCA. Thus, further research is required.

## Conclusions

In conclusion, we identified nine m6A-related lncRNAs with potential prognostic value in BLCA and developed a prognostic and predictive m6A-RLPS, which may be applied in the investigation of the molecular mechanisms involved in BLCA oncogenesis and the determination of the treatment efficacy in patients with BLCA.

## Supplementary Information

**Additional file 1: Table S1.** Demographic and clinicopathological characteristics of patients with bladder cancer (*n* = 410).

**Additional file 2: Table S2.** The 51 m6A-related prognostic lncRNAs.

**Additional file 3: Fig. S1.** Correlations between the selected immune checkpoints and 51 m6A-related lncRNAs. (a-h) Correlation between hub lncRNAs and CTLA-4, GAL9, LAG-3, PD-1, PD-L1, PD-L2, TIGIT, and TIM-3, respectively. *p < 0.05.

**Additional file 4: Table S3.** Clinicopathological characteristics of patients with BLCA in the training and validation cohorts.

**Additional file 5: Fig. S2.** Verification of the expression and survival differences of several lncRNAs among the m6A-RLPS based on the GEO data**.** Differential expression of KCNQ1OT1 in the (a) GSE31189, (b) GSE51493, and (c) GSE31684 cohorts. (d) Kaplan–Meier curves indicating different OS of patients with different expression levels of KCNQ1OT1.

**Additional file 6: Fig. S3.** Analysis of the m6A-RLPS stratified by risk level in the entire cohort. (a) Kaplan–Meier curves for the m6A-RLPS. (b) Distributions of risk scores, survival status, and relative lncRNA expressions. (c) ROC curves for predicting 1-, 3-, and 5-year OS rates.

**Additional file 7: Fig. S4.** Calibration curves of the risk score based on the nomogram. Calibration curves of the 3- and 5-year overall survival in the (a) training and (b) validation sets (bootstrap method, 1000 repetitions).

**Additional file 8: Table S4.** The relationship between risk score and survival in 33 kinds of tumors.

## Data Availability

Publicly available datasets were analyzed in this study. These data can be found here: https://portal.gdc.cancer.gov; https://www.ncbi.nlm.nih.gov/geo/; http://xena.ucsc.edu.

## Abbreviations

ACC: Adrenocortical carcinoma
AUC: Area under the curve
BLCA: Bladder cancer
BRCA: Breast invasive carcinoma
CESC: Cervical squamous cell carcinoma and endocervical adenocarcinoma
CHOL: Cholangiocarcinoma
C-index: Concordance index
COAD: Colon adenocarcinoma
DLBC: Lymphoid Neoplasm Diffuse Large B-cell Lymphoma
DSS: Disease-Specific Survival
ESCA: Esophageal carcinoma
GBM: Glioblastoma multiforme
GEO: Gene Expression Omnibus
GSEA: Gene set enrichment analysis
HNSC: Head and Neck squamous cell carcinoma
HR: Hazard ratio
ICIs: Immune checkpoint inhibitors
KICH: Kidney Chromophobe
KIRC: Kidney renal clear cell carcinoma
KIRP: Kidney renal papillary cell carcinoma
LAML: Acute Myeloid Leukemia
LASSO: Least absolute shrinkage and selection operator
LGG: Brain Lower Grade Glioma
LIHC: Liver hepatocellular carcinoma
lncRNAs: Long non-coding RNAs
LUAD: Lung adenocarcinoma
LUSC: Lung squamous cell carcinoma
m6A: N6-methyladenosine
m6A-RLPS: m6A-related lncRNA prognostic signature
MESO: Mesothelioma
mRNAs: Messenger RNAs
ncRNAs: Non-coding RNAs
OS: Overall survival
OV: Ovarian serous cystadenocarcinoma
PAAD: Pancreatic adenocarcinoma
PCPG: Pheochromocytoma and Paraganglioma
PRAD: Prostate adenocarcinoma
READ: Rectum adenocarcinoma
RNA-seq: RNA-sequencing
ROC: Receiver operating characteristic
SARC: Sarcoma
SKCM: Skin Cutaneous Melanoma
STAD: Stomach adenocarcinoma
TCGA: The Cancer Genome Atlas
TGCT: Testicular Germ Cell Tumors
THCA: Thyroid carcinoma
THYM: Thymoma
TICs: Tumor-infiltrating immune cells
TME: Tumor microenvironment
Tregs: Regulatory T cells
UCEC: Uterine Corpus Endometrial Carcinoma
UCS: Uterine Carcinosarcoma
UVM: Uveal Melanoma
