# Supplementary material for: An effective N6-methyladenosine-related long non-coding RNA prognostic signature for predicting the prognosis of patients with bladder cancer
Source: BMC Cancer. 2021 Nov 21;21:1256. doi: 10.1186/s12885-021-08981-4 (PMC8607649; doi:10.1186/s12885-021-08981-4)
Supplement: Supplementary file 1 — Additional file 1: Table S1. Demographic and clinicopathological characteristics of patients with bladder cancer (n = 410). [file 12885_2021_8981_MOESM1_ESM.docx]

**Table S1. Demographic and clinicopathological characteristics of patients with bladder cancer(n=410).**

| **Characteristics** | **No.** | **%** |
| --- | --- | --- |
| **Age(years)** |  |  |
| ≤65 | 161 | 39.27 |
| >65 | 249 | 60.73 |
| **Gender** |  |  |
| Female | 108 | 26.34 |
| Male | 302 | 73.66 |
| **Grade** |  |  |
| Low grade | 20 | 4.88 |
| High grade | 387 | 94.39 |
| Unknown | 3 | 0.73 |
| **Stage** |  |  |
| Stage I-II | 132 | 32.30 |
| Stage III-IV | 276 | 67.32 |
| Unknown | 2 | 0.49 |
| **T stage** |  |  |
| T0-T2 | 123 | 30.00 |
| T3-T4 | 254 | 61.95 |
| Unknown | 33 | 8.05 |
| **N stage** |  |  |
| N0 | 238 | 58.05 |
| N1-3 | 130 | 31.71 |
| Unknown | 42 | 10.24 |
| **M stage** |  |  |
| M0 | 195 | 47.56 |
| M1 | 11 | 2.68 |
| Unknown | 204 | 49.76 |
