# Supplementary material for: An effective N6-methyladenosine-related long non-coding RNA prognostic signature for predicting the prognosis of patients with bladder cancer
Source: BMC Cancer. 2021 Nov 21;21:1256. doi: 10.1186/s12885-021-08981-4 (PMC8607649; doi:10.1186/s12885-021-08981-4)
Supplement: Supplementary file 2 — Additional file 2: Table S2. The 51 m6A-related prognostic lncRNAs. [file 12885_2021_8981_MOESM2_ESM.docx]

**Table S2. The 51 m6A-related prognostic lncRNAs.**

| **m6A-related lncRNA** | **HR** | **HR.95L** | **HR.95H** | **P-value** |
| --- | --- | --- | --- | --- |
| **RAP2C-AS1** | 3.0340 | 1.3329 | 6.9064 | 0.0082 |
| **AC005479.1** | 2.2904 | 1.2739 | 4.1182 | 0.0056 |
| **KCNQ1OT1** | 2.1734 | 1.4070 | 3.3574 | 0.0005 |
| **AC025280.1** | 1.9757 | 1.2850 | 3.0377 | 0.0019 |
| **AP001469.1** | 1.9442 | 1.2012 | 3.1468 | 0.0068 |
| **AC097359.2** | 1.7970 | 1.1950 | 2.7021 | 0.0049 |
| **AC087286.2** | 1.4630 | 1.1054 | 1.9362 | 0.0078 |
| **AC012568.1** | 1.2462 | 1.0740 | 1.4461 | 0.0037 |
| **ATP1B3-AS1** | 1.2031 | 1.0842 | 1.3351 | 0.0005 |
| **THAP9-AS1** | 0.9378 | 0.8947 | 0.9828 | 0.0073 |
| **AC008735.2** | 0.9268 | 0.8759 | 0.9807 | 0.0084 |
| **LINC02604** | 0.9017 | 0.8461 | 0.9610 | 0.0014 |
| **PTOV1-AS2** | 0.8809 | 0.8219 | 0.9442 | 0.0003 |
| **TMEM147-AS1** | 0.8665 | 0.7888 | 0.9519 | 0.0028 |
| **ZNF436-AS1** | 0.8650 | 0.7785 | 0.9610 | 0.0069 |
| **AC022150.2** | 0.8626 | 0.7788 | 0.9555 | 0.0046 |
| **LINC01355** | 0.8298 | 0.7201 | 0.9562 | 0.0099 |
| **AC004148.1** | 0.8290 | 0.7454 | 0.9219 | 0.0005 |
| **AC074117.1** | 0.8283 | 0.7282 | 0.9422 | 0.0042 |
| **AL022328.2** | 0.8252 | 0.7280 | 0.9355 | 0.0027 |
| **AL022322.1** | 0.8203 | 0.7255 | 0.9275 | 0.0016 |
| **SNHG20** | 0.8190 | 0.7174 | 0.9349 | 0.0031 |
| **AC132872.3** | 0.7756 | 0.6557 | 0.9174 | 0.0030 |
| **MAP3K14-AS1** | 0.6951 | 0.5281 | 0.9149 | 0.0095 |
| **AC116914.2** | 0.6925 | 0.5760 | 0.8325 | 0.0001 |
| **ZNF32-AS2** | 0.6897 | 0.5345 | 0.8899 | 0.0043 |
| **AC097641.2** | 0.6569 | 0.4812 | 0.8967 | 0.0081 |
| **ZNF32-AS1** | 0.6518 | 0.4790 | 0.8869 | 0.0064 |
| **AC008735.4** | 0.6292 | 0.4432 | 0.8934 | 0.0096 |
| **AC104532.2** | 0.6161 | 0.4578 | 0.8291 | 0.0014 |
| **LINC00115** | 0.6012 | 0.4200 | 0.8605 | 0.0054 |
| **AC104564.3** | 0.5942 | 0.4363 | 0.8092 | 0.0010 |
| **AC010201.2** | 0.5790 | 0.3838 | 0.8734 | 0.0092 |
| **AC073534.2** | 0.5707 | 0.4100 | 0.7942 | 0.0009 |
| **AC012615.6** | 0.5612 | 0.3790 | 0.8310 | 0.0039 |
| **AC073575.4** | 0.5515 | 0.3720 | 0.8176 | 0.0030 |
| **IGBP1-AS1** | 0.4955 | 0.2917 | 0.8417 | 0.0094 |
| **Z84485.1** | 0.4807 | 0.2908 | 0.7946 | 0.0043 |
| **AL136295.2** | 0.4676 | 0.3113 | 0.7022 | 0.0002 |
| **AL161452.1** | 0.4412 | 0.2444 | 0.7963 | 0.0066 |
| **AC005306.1** | 0.4266 | 0.2386 | 0.7626 | 0.0041 |
| **AC021321.1** | 0.4016 | 0.2474 | 0.6519 | 0.0002 |
| **SPAG5-AS1** | 0.3952 | 0.2007 | 0.7782 | 0.0072 |
| **AC008764.8** | 0.3640 | 0.1703 | 0.7778 | 0.0091 |
| **AC068768.1** | 0.2867 | 0.1139 | 0.7216 | 0.0080 |
| **EHMT2-AS1** | 0.2768 | 0.1369 | 0.5598 | 0.0004 |
| **AC004076.2** | 0.2674 | 0.1105 | 0.6469 | 0.0034 |
| **AC020911.1** | 0.1963 | 0.0661 | 0.5834 | 0.0034 |
| **AC006160.1** | 0.1829 | 0.0533 | 0.6278 | 0.0069 |
| **AL138921.1** | 0.1806 | 0.0600 | 0.5441 | 0.0024 |
| **AC007686.3** | 0.1623 | 0.0438 | 0.6017 | 0.0065 |

m6A, N6-methyladenosine; lncRNA: long non-coding RNA; HR, hazard ratio.
