# Supplementary material for: An effective N6-methyladenosine-related long non-coding RNA prognostic signature for predicting the prognosis of patients with bladder cancer
Source: BMC Cancer. 2021 Nov 21;21:1256. doi: 10.1186/s12885-021-08981-4 (PMC8607649; doi:10.1186/s12885-021-08981-4)
Supplement: Supplementary file 3 — Additional file 3: Fig. S1. Correlations between the selected immune checkpoints and 51 m6A-related lncRNAs. (a-h) Correlation between hub lncRNAs and CTLA-4, GAL9, LAG-3, PD-1, PD-L1, PD-L2, TIGIT, and TIM-3, respectively. *p < 0.05. [file 12885_2021_8981_MOESM3_ESM.pdf]

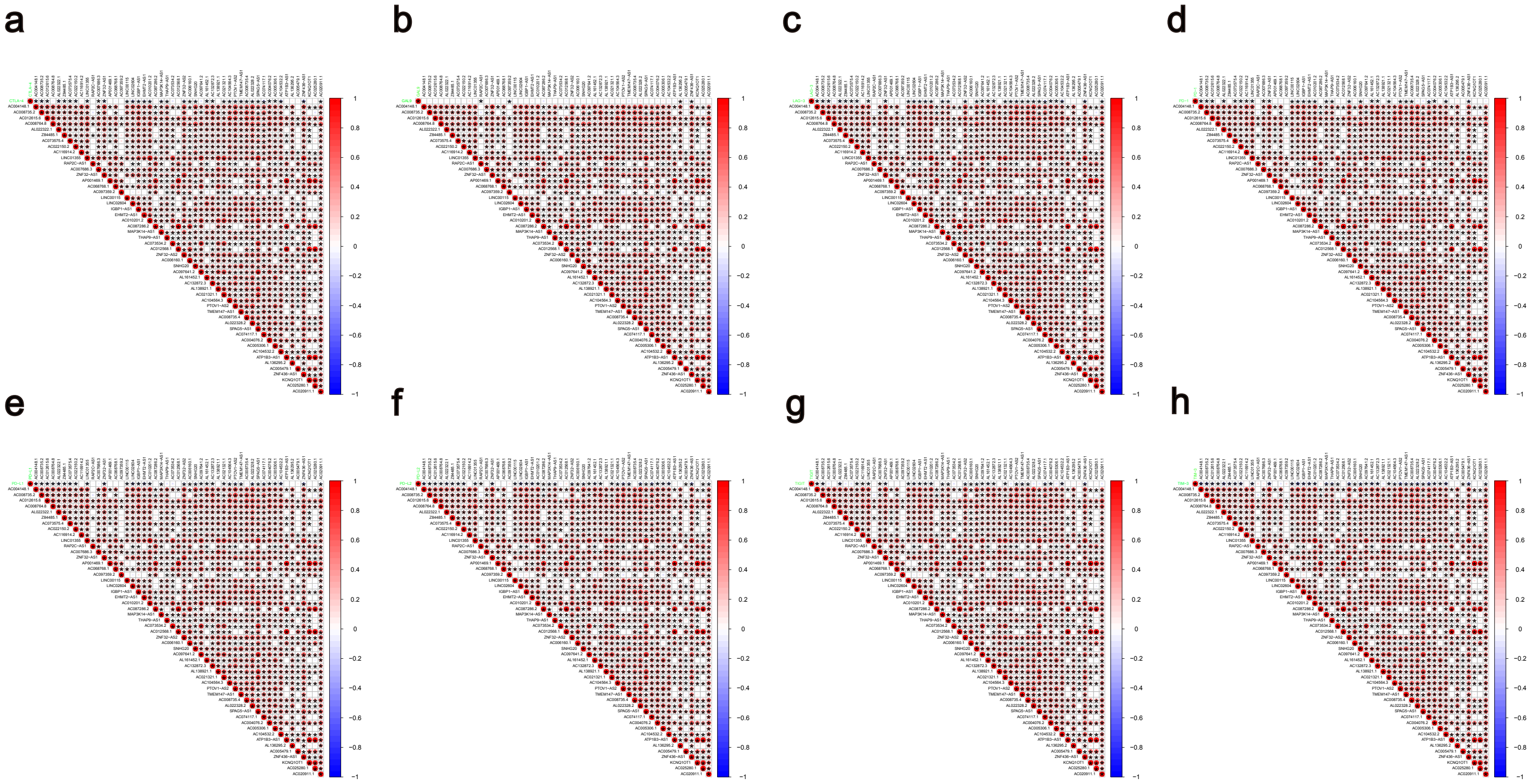

Additional file 3: Fig S1. Correlations between the selected immune checkpoints and 51 m6A-related lncRNAs. (a-h) Correlation between hub lncRNAs and CTLA-4, GAL9, LAG-3, PD-1, PD-L1, PD-L2, TIGIT, and TIM-3, respectively. *$p < 0.05$.
