# Supplementary material for: An effective N6-methyladenosine-related long non-coding RNA prognostic signature for predicting the prognosis of patients with bladder cancer
Source: BMC Cancer. 2021 Nov 21;21:1256. doi: 10.1186/s12885-021-08981-4 (PMC8607649; doi:10.1186/s12885-021-08981-4)
Supplement: Supplementary file 4 — Additional file 4: Table S3. Clinicopathological characteristics of patients with BLCA in the training and validation cohorts. [file 12885_2021_8981_MOESM4_ESM.docx]

**Table S3. Clinicopathological characteristics of BLCA patients in the training and validation cohorts.**

| **Features** | **Training cohort(n=203)** | **Validation cohort(n=200)** | **P-value** |
| --- | --- | --- | --- |
| **Age(years)** |  |  | 0.426 |
| ≤65 | 84(41.38%) | 75(37.50%) |  |
| >65 | 119(58.62%) | 125(62.50) |  |
| **Gender** |  |  | 0.480 |
| Female | 56(27.59%) | 49(24.50%) |  |
| Male | 147(72.41%) | 151(75.50%) |  |
| **Grade** |  |  | 0.864 |
| Low grade | 11(5.42%) | 9(4.50%) |  |
| High grade | 190(93.60%) | 190(95.00%) |  |
| Unknown | 2(0.99%) | 1(0.50%) |  |
| **Stage** |  |  | 0.144 |
| Stage I-II | 59(29.06%) | 71(35.50%) |  |
| Stage III-IV | 142(69.95%) | 129(64.50%) |  |
| Unknown | 2(0.99%) | 0(0%) |  |
| **T stage** |  |  | 0.743 |
| T0-T2 | 58(28.57%) | 64(32.00%) |  |
| T3-T4 | 128(63.05%) | 121(60.50%) |  |
| Unknown | 17(8.37%) | 15(7.50%) |  |
| **N stage** |  |  | 0.107 |
| N0 | 108(53.20%) | 126(63.00%) |  |
| N1-3 | 70(34.48%) | 58(29.00%) |  |
| Unknown | 25(12.32%) | 16(8.00%) |  |
