# Supplementary figures and images for: An effective N6-methyladenosine-related long non-coding RNA prognostic signature for predicting the prognosis of patients with bladder cancer
Source: BMC Cancer. 2021 Nov 21;21:1256. doi: 10.1186/s12885-021-08981-4 (PMC8607649; doi:10.1186/s12885-021-08981-4)

**a**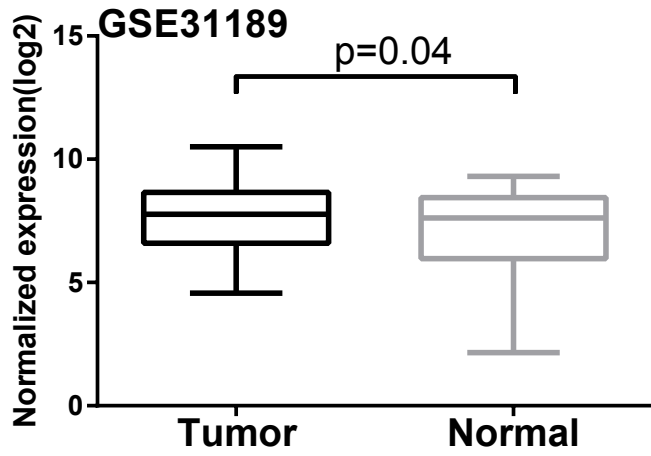**b**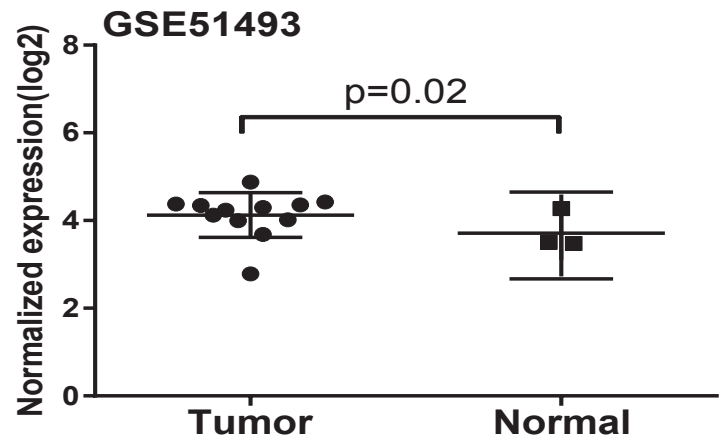**c**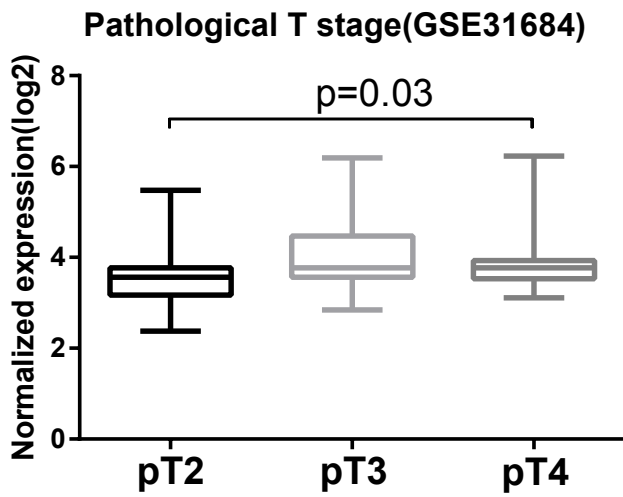**d**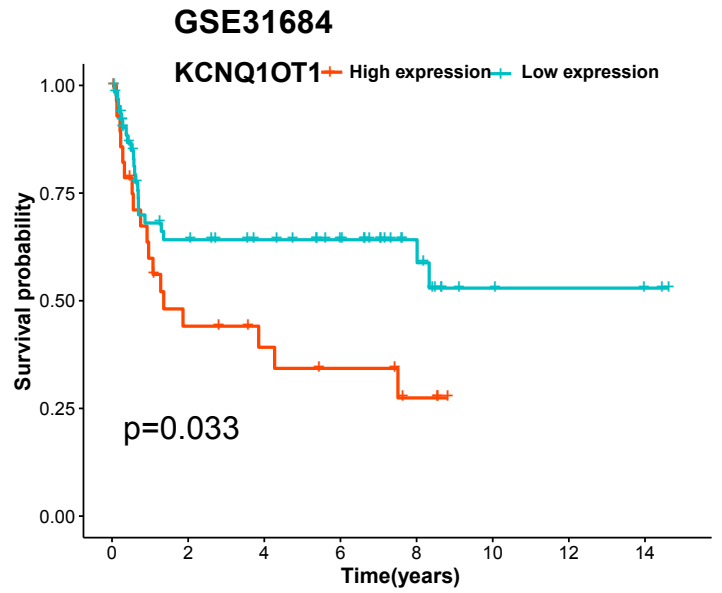

Supplement: Supplementary file 5 — Additional file 5: Fig. S2. Verification of the expression and survival differences of several lncRNAs among the m6A-RLPS based on the GEO data. Differential expression of KCNQ1OT1 in the (a) GSE31189, (b) GSE51493, and (c) GSE31684 cohorts. (d) Kaplan–Meier curves indicating different OS of patients with different expression levels of KCNQ1OT1. [file 12885_2021_8981_MOESM5_ESM.pdf]
