# Supplementary material for: An effective N6-methyladenosine-related long non-coding RNA prognostic signature for predicting the prognosis of patients with bladder cancer
Source: BMC Cancer. 2021 Nov 21;21:1256. doi: 10.1186/s12885-021-08981-4 (PMC8607649; doi:10.1186/s12885-021-08981-4)
Supplement: Supplementary file 6 — Additional file 6: Fig. S3. Analysis of the m6A-RLPS stratified by risk level in the entire cohort. (a) Kaplan–Meier curves for the m6A-RLPS. (b) Distributions of risk scores, survival status, and relative lncRNA expressions. (c) ROC curves for predicting 1-, 3-, and 5-year OS rates. [file 12885_2021_8981_MOESM6_ESM.pdf]

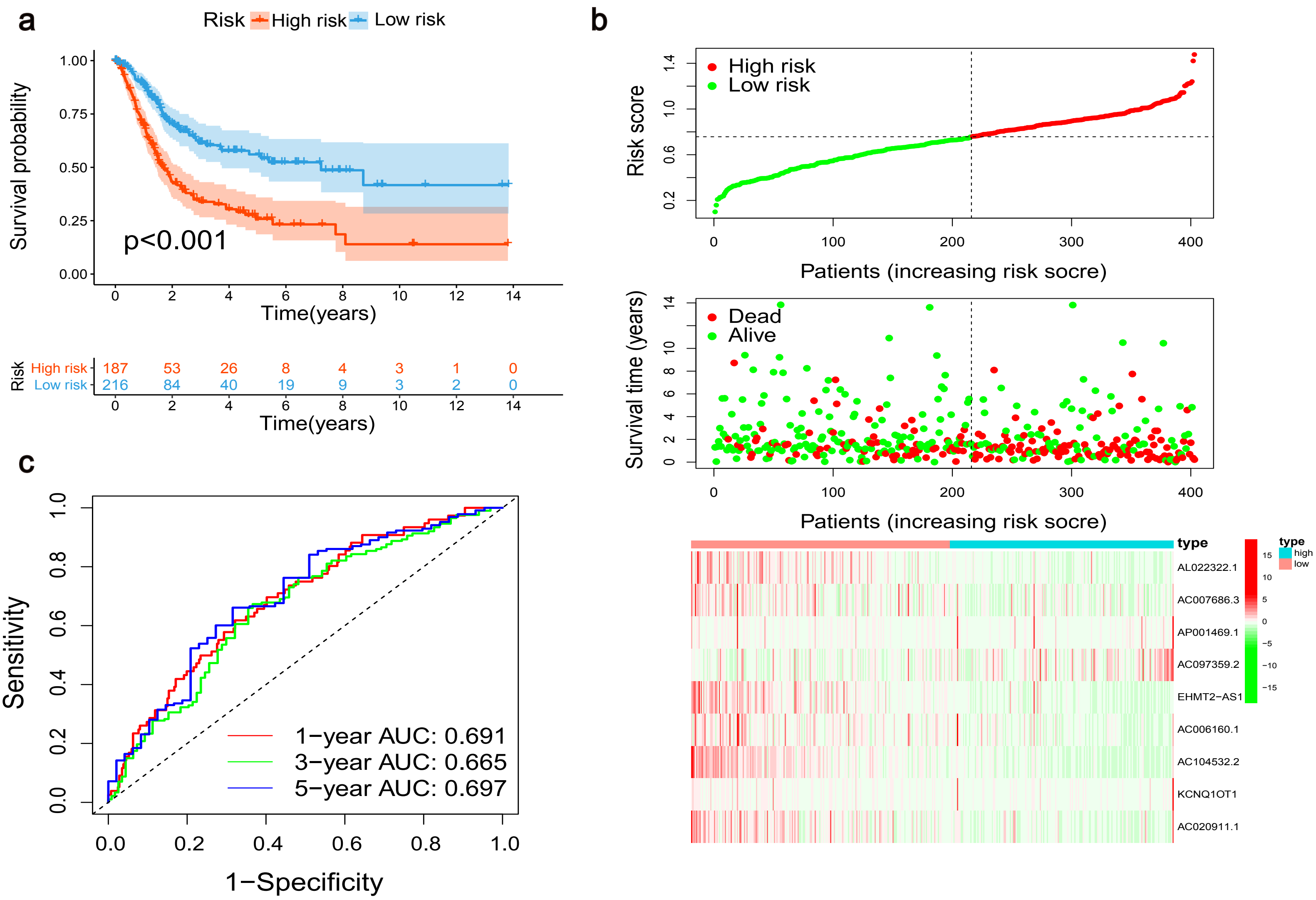

Additional file 5: Fig S2. Analysis of the m6A-RLPS stratified by risk level in the entire cohort. (a) Kaplan–Meier curves for the m6A-RLPS. (b) Distributions of risk scores, survival status, and relative lncRNA expressions. (c) ROC curves for predicting 1-, 3-, and 5-year OS rates.
