# Supplementary material for: An effective N6-methyladenosine-related long non-coding RNA prognostic signature for predicting the prognosis of patients with bladder cancer
Source: BMC Cancer. 2021 Nov 21;21:1256. doi: 10.1186/s12885-021-08981-4 (PMC8607649; doi:10.1186/s12885-021-08981-4)
Supplement: Supplementary file 7 — Additional file 7: Fig. S4. Calibration curves of the risk score based on the nomogram. Calibration curves of the 3- and 5-year overall survival in the (a) training and (b) validation sets (bootstrap method, 1000 repetitions). [file 12885_2021_8981_MOESM7_ESM.pdf]

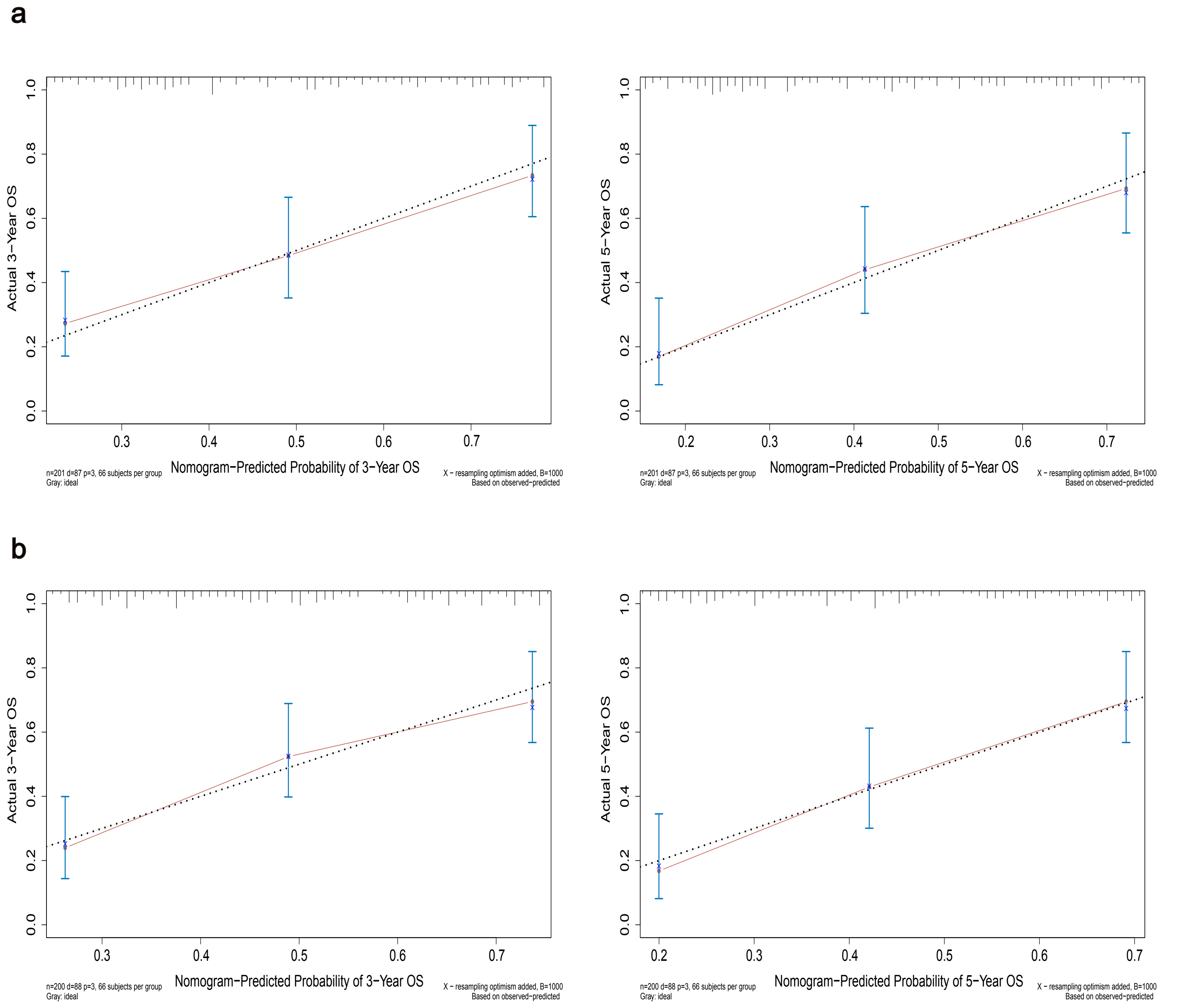

Additional file 6: Fig S3. Calibration curves of the risk score based on the nomogram. Calibration curves of the 3- and 5-year overall survival in the (a) training and (b) validation sets (bootstrap method, 1000 repetitions).
